# DOES INTERNET USE MODERATE THE RURAL–URBAN DISPARITIES IN HEALTH AMONG BRAZILIAN OLDER ADULTS?

**DOI:** 10.1093/geroni/igae098.2521

**Published:** 2024-12-31

**Authors:** Xiayu Chen, Tania Rodriguez, Rachel Wu, Flávia Andrade

**Affiliations:** University of Illinois Urbana-Champaign, Champaign, Illinois, United States; University of California Riverside, Riverside, California, United States; University of California Riverside, Riverside, California, United States; University of Illinois at Urbana Champaign, Urbana, Illinois, United States

## Abstract

Although prior research has noted the benefits of Internet use on physical and mental health among older adults in developed countries, its impact within less developed countries, particularly in Latin America concerning rural-urban disparities, is poorly understood. This study investigated the health disparities between rural and urban older adults in Brazil and examined how Internet use moderated these disparities. Data from the second wave of the Brazilian Longitudinal Study of Aging with 9,751 adults aged 50 and above were analyzed. The outcome variables include the number of chronic conditions, limitations in basic and instrumental activities of daily living (ADL & IADL), depressive symptoms and cognitive functioning. Using a series of weight-adjusted multivariate linear regression analyses, we found that compared to the older adults who lived in rural areas, urban residents tended to have more chronic conditions, IADL disabilities, higher depressive symptoms, but better cognitive functioning. A significant interaction between Internet use and rural-urban residency was identified for chronic conditions (β=0.72, 95% CI= 0.55-0.94), cognitive functioning (β=0.85, 95% CI=0.73-0.99), and depressive symptoms (β=1.40, 95% CI=1.04-1.89), indicating that Internet use mitigated the rural-urban disparity in chronic conditions and cognitive functioning, while it increased the disparity in depressive symptoms. The study’s findings underscore the potential of Internet use as a means to reduce health disparities among older adults in Brazil. Future interventions could include targeted digital inclusion strategies tailored to address the socioeconomic challenges in rural areas to leverage the benefits of the Internet.

